# Longitudinal analysis of symptom-based clustering in patients with primary Sjogren’s syndrome: a prospective cohort study with a 5-year follow-up period

**DOI:** 10.1186/s12967-021-03051-6

**Published:** 2021-09-19

**Authors:** Jennifer Jooha Lee, Young Jae Park, Misun Park, Hyeon Woo Yim, Sung Hwan Park, Seung-Ki Kwok

**Affiliations:** 1 Division of Rheumatology, Department of Internal Medicine, College of Medicine, Seoul St. Mary’s Hospital, The Catholic University of Korea, Seoul, 06591 Republic of Korea; 2 Department of Preventive Medicine, College of Medicine, The Catholic University of Korea, Seoul, 06591 Republic of Korea

**Keywords:** Cluster analysis, Latent class analysis, Sjogren’s syndrome

## Abstract

**Background:**

Sjogren’s syndrome (SS) is a heterogenous disease with various phenotypes. We aimed to provide a relevant subclassification based on symptom-based clustering for patients with primary (p) SS.

**Methods:**

Data from patients in a prospective pSS cohort in Korea were analysed. Latent class analysis (LCA) was performed using patient reported outcomes, including pain, fatigue, dryness, and anxiety/depression. Clinical and laboratory differences between the classes were analysed. Latent transition analysis (LTA) was applied to the longitudinal data (annually for up to 5 years) to assess temporal stability of the classifications.

**Results:**

LCA identified three classes among 341 patients with pSS (i.e., ‘high symptom burden’, ‘dryness dominant’, ‘low symptom burden’). Each group had distinct laboratory and clinical phenotypes. LTA revealed that class membership remained stable over time. Baseline class predicted future salivary gland function and damage accrual represented by a Sjogren’s syndrome disease damage index.

**Conclusion:**

Symptom-based clustering of heterogenous patients with primary Sjogren’s syndrome provided a relevant classification supported by temporal stability over time and distinct phenotypes between the classes. This clustering strategy may provide more homogenous groups of pSS patients for novel treatment development and predict future phenotypic evolvement.

**Supplementary Information:**

The online version contains supplementary material available at 10.1186/s12967-021-03051-6.

## Background

Sjogren’s syndrome (SS) is a systemic autoimmune disease characterised by sicca symptoms associated with lymphocytic infiltrates of affected glands [1]. Some patients with SS suffer from various extra glandular manifestations (e.g., arthralgia/arthritis, Raynaud’s phenomenon, peripheral neuropathy, interstitial lung disease) [2]. Therefore, between-group heterogeneity in phenotype and severity occurs in patients with SS.

Given the lack of pathogenesis-targeted therapies, treatment for SS is mainly focused on symptom relief [3]. Novel biologics, such as rituximab, fail to meet the primary endpoint of clinical trials [4]. This result appears to occur partly because inclusion criteria were not appropriate to select a group of patients homogenous enough to have similar responses. On the other hand, some trials (e.g., abatacept trial) [5] used the high European League Against Rheumatism (EULAR) Sjogren’s syndrome disease activity index (ESSDAI) as inclusion criteria and found that ESSDAI scores fail to improve more so than scores generated using a placebo. It appears that heterogenous groups of patients had high ESSDAI scores but different phenotypes within groups. These trials might have been successful if they were performed using more homogenous groups of patients.

Tarn et al. found that symptom-based stratification of patients with SS identified four distinct subgroups with unique pathobiological endotypes [6]. The authors re-analysed data from two large clinical trials, JOQUER [7] and TRACTISS [8], and found that hydroxychloroquine or rituximab, respectively, were efficacious in specific subgroups of patients. Stratification was performed based on baseline characteristics of an existing United Kingdom Primary Sjogren’s Syndrome Registry cohort [9]. They also performed an external validation study using external cohorts and found good performance of the classification system. Nevertheless, to apply this stratification clinically, it is important that class membership remains stable over the time so that stratification can be performed at any time during the disease course. Clustering methods will also be more advantageous if stratification could predict future disease status.

Based on these, we investigated whether symptom-based clustering performed well enough to provide relevant classes in a population of Korean patients with pSS. We also sought to determine if class membership had temporal stability during a 5-year follow-up period. Finally, we examined whether the initial class predicted future disease status in terms of salivary flow rate (SFR) and Sjogren’s syndrome damage index (SSDDI) results.

## Methods

### Study population

In this study, all enrolled patients with pSS were Korean Initiative Sjogren’s Syndrome (KISS) participants that were recruited at Seoul St. Mary’s Hospital. The KISS was founded in 2013 with the aims to establish a nationwide prospective cohort database that contained overall clinical data and samples from patients with pSS, and to develop diagnostic and treatment tools for pSS. Informed consent was obtained from all patients according to Declaration of Helsinki principles. This study was approved by the Institutional Review Board of Seoul St. Mary’s Hospital (KC13ONMI0646). All data were collected and managed using the Clinical Research and Trial Management System (Korea National Institutes of Health, Korea Centers for Disease Control and Prevention). Recruitment began in October 2013 at Seoul St. Mary’s Hospital, which is a tertiary care university hospital and referral center in Seoul, Korea. Diagnosis of pSS was made based on American-European Consensus Group criteria for pSS or 2012 provisional American College of Rheumatology criteria. By January 2016, the database included 321 pSS patients from Seoul St. Mary’s Hospital. Enrollment was suspended by that time, and patients have subsequently been followed up with annually.

### Statistical methods

Latent class analysis (LCA) was used for clustering. The variables selected for clustering included components of the EULAR SS Patient Reported Index (ESSPRI) and the EQ-5D. Visual analogue scales (VASs) of pain, fatigue, and dryness, with values from 0–10 derived from ESSPRI. Anxiety/depression was examined using the 5-Likert scales from the EQ-5D. Usually, binary variables are applied when using LCA. Definition of variables such as pain > 3, dryness > 5, and fatigue > 7 were made according to baseline median values. A clinically meaningful value ≥ 3 was used for anxiety/depression, (Additional file 1: Table S1). Model fitness was measured using Akaike’s information criterion, Bayes information criterion (BIC), G-Squared, entropy, and log-likelihood [10] results (Additional file 1: Table S2).

Latent transition analysis (LTA) is a longitudinal version of LCA. LTA provides class membership probabilities at each time point, and probabilities of transitioning to a different class over time [11]. Results for fit statistics for LTA are presented in Additional file 1: Table S3.

Clinical and laboratory parameters were compared between classes. Continuous variables were compared using Kruskal Wallis tests with post-hoc analysis. Categorical variables were compared using chi-square tests. A value of P < 0.05 was considered to be significant. Statistical analysis was performed using SAS software (version 9.4; SAS institute, Cary, NC, USA) with PROC LCA and PROC LTA downloaded from https://www.methodology.psu.edu/downloads/proclcalta/, and IBM SPSS Statistics for Windows (version 24; IBM corp., Armonk, NY, USA).

## Results

### Latent class analysis identifies three classes in patients with pSS

LCA was performed using components of ESSPRI and EQ-5D from baseline to 5 years of follow-up. Supplementary table 4 presents the results for numbers of subjects during the follow-up and their VAS results for the ESSPRI and depression/anxiety scales. Calculation of fit statistics found that three-class clustering had the most relevant performance, which was represented by low BIC and high entropy values (Additional file 1: Table S2).

The class 1 (66 out of 321) group had ‘dryness dominant’ characteristics. Patients in class 1 had low levels of pain, but suffered from dryness and fatigue comparable to patients in class 2 (Table 1). Class 2 (134 out of 321) was characterised as ‘high symptom burden.’ Patients in class 2 had high VAS scores for all four components. Class 3 (121 out of 321) patients had relatively mild symptoms in all four areas, compared with the other two groups.

**Table 1:** Baseline demographic and clinical characteristics of each class

	Class 1	Class 2	Class 3	*P*
	Dryness dominant	High symptom burden	Low symptom burden	
	(n = 66)	(n = 134)	(n = 121)	
Pain	0 [0–3]	5 [4–7]	2 [0–3]	< 0.001
Fatigue	5 [4–6]	7 [6–8]	5 [3–5.5]	< 0.001
Dryness	8 [8–10]	8 [7–9]	5 [5–7]	< 0.001
Anxiety/depression	2 [1, 2]	3 [2, 3]	2 [1, 2]	< 0.001
Age	55 [47.75–60]	51 [42.75–60]	54 [43–59.5]	0.224
Female (%)	65 (98.5%)	133 (99.3%)	119 (98.3%)	0.789
Disease duration (mo)	23.5 [2.3–56.2]	22.7 [1.6–61.1]	10.4 [1.3–44.5]	0.162
uSFR (mL/5 min)	n = 46 0.1 [0–0.25]	n = 97 0.1 [0–0.4]	n = 89 0.25 [0.1–0.5]	< 0.001
sSFR (mL/5 min)	n = 40 1.5 [0.85–4.38]	n = 83 2.8 [1.5–5]	n = 70 2.75 [1.15–6.05]	0.091
Xerostomia Inventory	40.5 [31.75–44]	40.5 [34–46]	32 [25–39]	< 0.001
Anti Ro positivity	56/66 (84.8%)	113/134 (84.3%)	105/121 (86.8%)	0.851
IgG	1634 [1310–2038.5]	1612 [1378–2052]	1520 [1329–1816.5]	0.155
ACPA positivity	5/66 (7.6%)	11/124 (8.9%)	12/117 (10.3%)	0.826
RF positivity	43/64 (67.2%)	79/129 (61.2%)	79/120 (65.8%)	0.644
Cryoglobulin positivity	n = 63 2 (3.2%)	n = 130 1 (0.8%)	n = 113 0 (0%)	0.116
Complement 3	87 [78–99.5]	94 [80–101]	93 [82–102]	0.163
Complement 4	22.7 [18.8–29.8]	21.3 [17.4–25.6]	22.3 [18.4–26.3]	0.398
Schirmer's test (OD)	n = 62 3 [1.75–5]	n = 112 4.5 [2–8]	n = 104 3.5 [2.25–7]	0.006
OSS (OD)	4 [2–6.25]	3 [1–5]	3 [1–5]	0.004
OSDI	35 [21.5–56.5]	48 [32–64]	28 [14–43]	< 0.001
EGM				
Arthralgia/arthritis	13(19.7%)	81 (60.4%)	50 (41.3%)	< 0.001
Raynaud	13 (21.3%)	29(21.6%)	19 (15.7%)	0.495
Lymphadenopathy	8 (12.1%)	24 (17.9%)	17 (14.0%)	0.512
Pulmonary involvement	2 (3.0%)	5 (3.7%)	6 (5%)	0.783
Cutaneous involvement	6 (9.1%)	28 (20.9%)	13 (10.7%)	0.027
Liver involvement	4 (6.1%)	8 (6%)	3 (2.5%)	0.358
Kidney involvement	0	6(4.5%)	1 (0.8%)	0.055
Peripheral neuropathy	3 (4.5%)	25 (18.7%)	7 (5.8%)	0.001
CNS	0	3(2.2%)	1 (0.8%)	0.356
Autoimmune thyroid disease	15(22.7%)	17(12.7%)	18(15%)	0.179
Fibromyalgia	1 (1.5%)	12 (9.0%)	3 (2.5%)	0.021
ESSPRI	5 [4.3–5.7]	6.7 [6–7.7]	4 [3–4.7]	< 0.001
ESSDAI	3 [1–6]	4 [2–8]	3 [1–5.75]	0.03
Articular	0	0[0–1]	0	0.004
PNS	0	0	0	0.027
Biological	1 [0–2]	1 [0–2]	0 [0–1]	0.041
Pt GA	72 [56.75–85.25]	73 [62–83.25]	52 [33–63.5]	< 0.001
Phy GA	30 [15–45]	39 [20–50]	30 [ 13.25–44.75]	0.014
SSDDI	3 [2, 3]	2 [2, 3]	2 [2, 3]	0.014
Hydroxychloroquine	41/66 (62.1%)	85/134 (63.4%)	77/121 (63.6%)	0.977
Methotrexate	0/66	6/134	2/121	0.137
Azathioprine	2/66	4/134	1/121	0.434
Corticosteroid	24/66 (36.4%)	63/134 (47.0%)	33/121 (27.3%)	0.0049
NSAID	3 (4.5%)	25 (18.7%)	16 (13.2%)	0.024
Pilocarpine	54/66 (81.8%)	115/134 (85.8%)	90/121 (74.4%)	0.067
Pilocarpine,dose (mg)	10 [5–10]	7.5 [5–10]	6.25 [2–7.5]	0.013
Salivary siglec-5 (pg/mL)	(n = 35) 4210 [1232.5–9085.9]	(n = 69) 978.3 [213.5–3181.4]	(n = 63) 925.1 [29.7–3450.6]	0.001

Data are expressed as median [interquartile ranges] values

*uSFR* unstimulated salivary flow rate, *sSFR* stimulated salivary flow rate, *IgG* immunoglobulin G, *CNS* central nervous system, *ESSPRI* Eular Sjogren's syndrome patient reported index, *ESSDAI* Eular Sjogren's syndrome disease activity index, *PtGA* pateint global assessment, *Phy* GA physician global assessment, *SSDDI* Sjogren's syndrome disease damage index

### Different phenotypes according to class

With regard to the endophenotype of each group, no between-class differences in age or disease duration were found (Table 1).

As expected, unstimulated (u)SFRs were lower in the dryness dominant, high symptom burden groups (class 1: 0.1 [0–0.25], class 2: 0.1 [0–0.4], class 3: 0.25 [0.1–0.5], P < 0.001). Accordingly, the xerostomia inventory scores were higher in these two groups (class 1: 40.5 [31.75–44], class 2: 40.5 [34–46], class 3: 32 [25–39], P < 0.001). The dryness dominant group had the worst objective eye parameter results (Schirmer’s test [P = 0.006], ocular staining score [P = 0.004]). Autoantibody profiles were not different between classes. However, low C3 level was more frequently found in dryness dominant group (24.6% (dryness dominant) vs 18.5% (high symptom burden) vs 10.7% (low symptom burden, P = 0.041). Cryoglobulin positivity tended to be higher in dryness dominant group (3.2% vs 0.8% vs 0%, P = 0.116) although it didn’t reach the statistical significance. Joint involvement is the most common extra glandular manifestation and class 2 patients had a significantly higher frequency of arthralgia/arthritis (class 1: 19.7%, class 2: 60.4%, class 3: 41.3%, P < 0.001), which explains the high pain VAS results in this group (class 1: 0 [0–3], class 2: 5 [4–7], class 3: 2 [0–3], P < 0.001). Cutaneous involvement (class 1: 9.1%, class 2: 20.9%, class 3: 10.7%, P = 0.027) and peripheral neuropathy (class 1: 4.5%, class 2: 18.7%, class 3: 5.8%, P = 0.001) was also more common in class 2. The frequency of fibromyalgia was higher in class 2 patients than other classes. The ESSDAI value was significantly higher in class 2, with significant differences in joint (P = 0.004), peripheral nervous system (P = 0.027), and biological domain (P = 0.041) results. Accordingly, patients in class 2 were more frequently treated using steroids (class 1: 36.4%, class 2: 47%, class 3: 27.3%, P = 0.0049)) and NSAIDs (class 1: 4.5%, class 2: 18.7%, class 3: 13.2%, P = 0.024).

### Temporal stability of classification determined using latent transition analysis

**Fig. 1:**
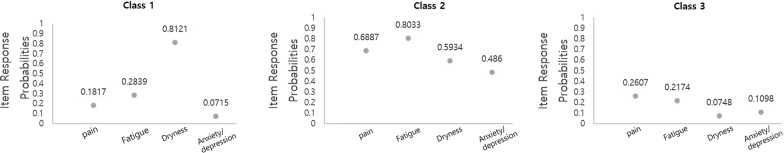
Item response probabilities at all times. Item response probabilities are depicted for each variable

**Fig. 2:**
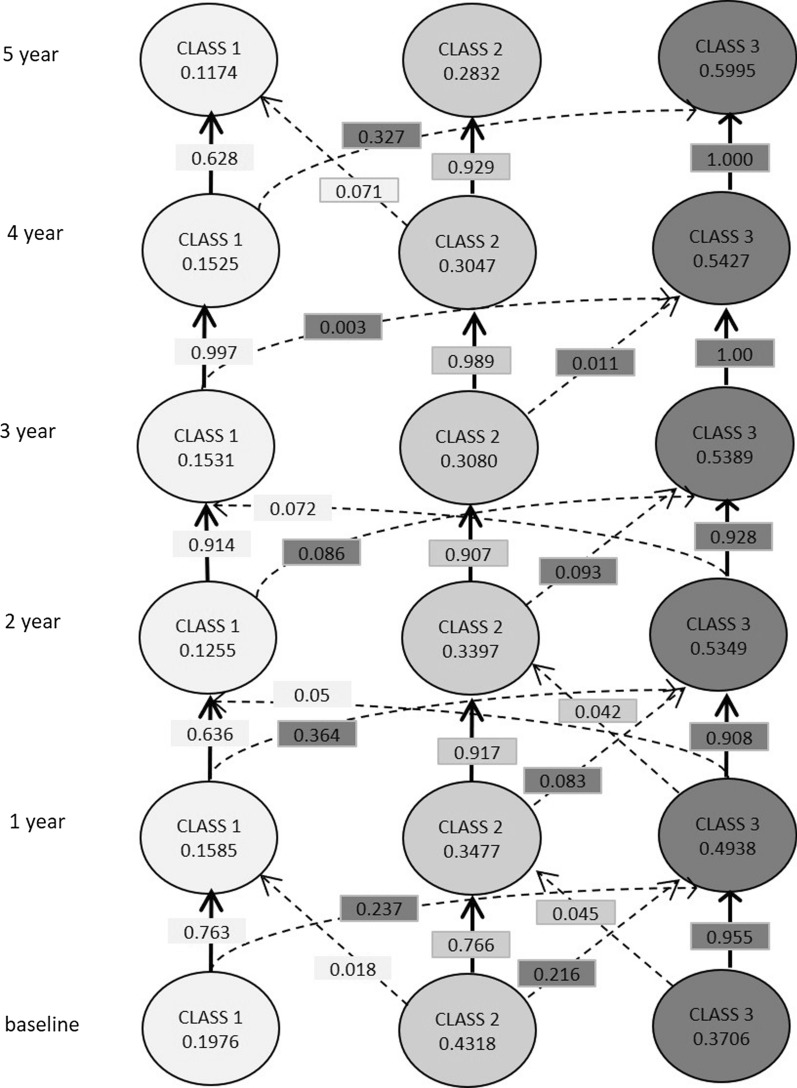
Transition probability matrix. Latent status prevalence and probabilities of transitioning into specific classes are depicted

Next, we performed LTA to investigate if class membership remained stable over time. Results for latent status and item response probabilities at all times are presented in Table 2 and Fig. 1. High VAS values for pain, fatigue, and anxiety/depression conferred a high probability to be classified as class 2. Dryness was highly associated with class 1. The results for transition probabilities indicated temporal stability of membership (Fig. 2). Patients with high symptom burden (class 2) tended to remain in the same class with annual transition probabilities more than 0.9, except during the initial 1-year period. Similarly, patients with low symptom burden (class 3) hardly moved to other classes (i.e., transition probability of nearly 1.0). The dryness dominant population (class 1) had a different trend compared with the other two groups. Patients in class 1 often experienced transition to class 3 (low symptom burden), with transition probabilities from 0.003 to 0.364. However, they did not move to the high symptom burden group (class 2) throughout the follow-up period.

**Table 2:** Latent status prevalence and item response probabilities

	Class 1	Class 2	Class 3
Latent status prevalence			
Baseline	0.1976	0.4318	0.3706
1 year	0.1585	0.3477	0.4938
2 years	0.1255	0.3397	0.5349
3 years	0.1531	0.308	0.5389
4 years	0.1525	0.3047	0.5427
5 years	0.1174	0.2832	0.5995
Item response probabilities (all times)			
Pain	0.1817	0.6887	0.2607
Fatigue	0.2839	0.8033	0.2174
Dryness	0.8121	0.5934	0.0748
Anxiety/depression	0.0715	0.486	0.1098

### Predictive ability of baseline classes

After verifying temporal stability of the classes, we examined whether initial class predicted deterioration associated with pSS. We compared the ESSPRI, SFR, SSDDI values on the last visits between those at baseline for each class. The median follow-up period was 4 years. We found significant differences in ESSPRI (P < 0.001), uSFR (P = 0.004), and SSDDI (P = 0.014) values (Table 3).

**Table 3:** Last follow-up values of SFR, ESSPRI, SSDDI according to baseline class

Last follow-up	Baseline			*P*
	Class 1	Class 2	Class 3	
uSFR (mL/5 min)	0.3 [0.1–0.575]	0.2 [0.025–0.475]	0.5 [0.15–1.2]	0.004
ESSPRI	4 [3–4.7]	5.7 [4–6.7]	3 [2–4.3]	< 0.001
SSDDI	3[2, 3]	3[2, 3]	2 [1–3]	0.014

Data are presented as median [interquartile range] values

*uSFR* unstimulated salivary flow rate, *ESSPRI* European Sjogren's syndrome patient reported index, *SSDDI* Sjogren's syndrome disease damage index

The low symptom burden group (class 3) continued to have low ESSPRI scores (3 [2–4.3] and relatively preserved uSFRs (0.5 [0.15–1.5]mL/5 min). Baseline class 2 patients still had the highest ESSPRI scores (5.7 [4–6.7]) and SSDDI values (3 [2–3]) were higher than that of patients in class 3. At baseline, uSFR was not different between classes 1 and 2, but the follow-up uSFR result was higher in patients in class 1. The SSDDI was higher in class 1 at baseline, but class 2 patients had higher SSDDIs at the last follow-up. These results suggested that initial symptom-based classification predicted future disease status at follow-up and supported the clinical relevance of the classification method.

## Discussion

In this study, we used symptom-based clustering and LCA to subclassify patients with pSS. Clustering revealed three classes with distinct endotypes and LTA revealed temporal stability of membership during and up to 5 years of follow-up. Baseline membership predicted future SFR and SSDDI results. This result suggested that the initial class determined different disease evolvement at the last follow-up.

The three latent classes identified were designated as ‘dryness dominant’, ‘high symptom burden’, and ‘low symptom burden’ groups. This nomenclature was originally derived from the Tarn et al. [6] report, which was the first to suggest the use of four symptom-based clusters in patients with pSS. The authors used the anxiety/depression scale from the hospital anxiety and depression scale (total score ranges from 0 to 42), instead of the EQ-5D scale. This difference may explain the difference in the numbers of classes between the two studies. In addition to the number of classes—3 compared with 4-, ESSDAI and medications were different between the classes in our study, which was not the case in the previous study. Contrary to the higher lymphoma prevalence along with β2-microglobulin and CXCL13 level in the dryness dominant group observed in the previous study, we could not find any difference in β2-microglobulin level. However, the only lymphoma patient in our cohort was classified to dryness dominant group which showed the highest cryoglobulin positivity—a risk factor for lymphoma in pSS consistent with the previous report. Therefore, patients in the same class seemed to have basically similar characteristics in both studies, which suggested that symptom-based clustering performed well regardless of ethnicity. A major strength of our classification criteria compared to the previous study is that the LCA method we used in the current permits LTA analysis which showed the temporal stability of a cluster over time, in addition to the use of cross-sectional clustering analysis. And the questionnaire for the classification is more simple.

Each class had distinct clinical and laboratory parameters associated with pSS. Nevertheless, symptom variables might not be objective parameters associated with the pathogenic mechanism of pSS. Subclassification of patients with pSS has focused on molecular signatures that appear to be more associated with pathogenesis of the disease [2, 12–15]. James et al. found that transcriptional modules identified three clusters with differences in interferon, inflammation modules, and molecules, such as CXCL10, CXCL9, and BAFF [16]. This classification that uses molecular features correlates well with systemic involvement represented by ESSDAI. However, other phenotypical differences do not seem to be affected by these molecular features. We found the same optimal performance of symptom-based clustering as the previous study, and verified its relevance and temporal stability. These results indicated this approach may be valid to subclassify patients with pSS. We also found that there was a significant difference in salivary siglec-5 results between the classes. We previously reported salivary siglec-5 as a biomarker for pSS diagnosis; it is negatively correlated with SFR and positively correlated with serum IgG [17]. Although baseline uSFR and IgG levels were not different, salivary siglec-5 was significantly higher in patients in class1 than in patients in class 2 (class 1: 4210 [1232.5–9085.9], class 2: 978.3 [213.5–3181.4] pg/mL, P = 0.001) (Table 1)). The differences might result from ‘latent’ differences between the two classes.

Precision medicine is one of the most interesting topics in the current medical field. Appropriate classification of patients cannot be over-emphasised for its value in specific application of unique and efficient treatment strategies. Therefore, the use of clustering has been suggested to be applied to many other diseases, including asthma [18–20], sepsis [21], and cardiovascular diseases [22]. With regard to rheumatologic diseases, studies have used different statistical methods for clustering systemic lupus erythematosus [23], systemic sclerosis [24], and IgG4-related disease [25]. These studies classified heterogenous groups of patients into more homogenous subgroups to better understand disease course and underlying mechanisms.

We used LCA for cluster analysis instead of the hierarchical analysis, which has been widely used in previous studies. One advantage of hierarchical analysis is that it displays a dendrogram, which allows for easy visual presentation of results [26]. An advantage of LCA is that it has been used as confirmatory analysis to reproduce results performed using k-means clustering and has been evaluated for use in person-centered analysis [27]. Using LCA, we performed LTA of longitudinal data as well.

From different perspectives, the temporal stability of this classification approach is favorable to explain the possible disease course of patients. For example, a patient with a low symptom burden (class 3) at baseline has a high chance of staying in that class during the follow-up period. The analysis of our longitudinal data indicated that it was not likely that he or she would experience high disease activity. Therefore, physicians might interpret the Sjogren’s syndrome disease entity, which can affect all systems of the body, as slow evolving and as one that largely results in mild clinical manifestations. To confirm this hypothesis, more long-term data is needed.

This study had some limitations. First, the number of patients in the study population was small and only included Korean patients with pSS from a single center. In these patients, systemic involvement was not frequent or severe. However, we obtained similar results in a previous study [6], which suggests that symptom-based clustering performs well in general. Second, symptom-based stratification is not based on variables associated with the pathogenesis itself. However, as previously mentioned, this method may identify the latent class of patients with pSS. Third, predictions of future ESSPRI, SFR, and SSDDI values were not derived from a model that adjusted for potential confounding variables. The ESSPRI results were expected because class remained stable over time, and the initial class with low symptom burden had low ESSPRI scores during the follow-up periods. The finding of significant differences in SFRs and SSDDIs at the last follow-up, according to initial class membership, conferred more value on the clustering method used. Currently, we are developing a regression model to predict class membership in another patient cohort, which aims to further validate the potential application of this classification method.

## Conclusions

Symptom-based clustering of heterogenous pSS patients provided a relevant classification that is supported by temporal stability over time and clearly distinct phenotypes between classes. This clustering strategy may identify more homogenous subgroups of patients with pSS, to aid in novel treatment development and to predict future phenotypic evolvement.

## Supplementary Information

**Additional file 1:Table S1.** Definition of variables for latent class analysis. **Table S2.** Fit statistics for latent class analysis. **Table S3.** Fit statistics for latent transition analysis. **Table S4.** Variables for latent class analysis performed annually from baseline.

## Data Availability

The datasets generated and/or analysed during the current study are available from the corresponding author on reasonable request.

## Abbreviations

ESSDAI: European League Against Rheumatism (EULAR) Sjogren’s syndrome disease activity index
ESSPRI: EULAR SS Patient Reported Index
LCA: Latent class analysis
LTA: Latent transition analysis
pSS: Primary Sjogren’s syndrome
SFR: Salivary flow rate
SSDDI: Sjogren’s syndrome damage index
