# Supplementary material for: Longitudinal analysis of symptom-based clustering in patients with primary Sjogren’s syndrome: a prospective cohort study with a 5-year follow-up period
Source: J Transl Med. 2021 Sep 19;19:394. doi: 10.1186/s12967-021-03051-6 (PMC8451081; doi:10.1186/s12967-021-03051-6)
Supplement: Supplementary file 1 — Additional file 1:Table S1. Definition of variables for latent class analysis. Table S2. Fit statistics for latent class analysis. Table S3. Fit statistics for latent transition analysis. Table S4. Variables for latent class analysis performed annually from baseline. [file 12967_2021_3051_MOESM1_ESM.docx]

**Supplementary table 1 Definition of variables for latent class analysis**

| Pain | Fatigue | Dryness | Anxiety/  depression |
| --- | --- | --- | --- |
| >3 (baseline median) | >5 (baseline median) | >7 (baseline median) | ≥3 |

**Supplementary table 2 Fit statistics for latent class analysis.**

|  | Latent class | Log-likelihood | BIC | aBIC | AIC | G SQUARED | Entropy | Log-likelihood |
| --- | --- | --- | --- | --- | --- | --- | --- | --- |
| Baseline | 1 | -854.663 | 90.771 | 78.084 | 75.686 | 67.686 | 1 | -665.163 |
|  | 2 | -824.326 | 58.954 | 30.408 | 25.011 | 7.011 | 0.477 | -642.994 |
|  | 3 | -821.099 | 81.357 | 36.951 | 28.557 | 0.557 | 0.617 | -642.994 |
|  | 4 | -820.83 | 109.675 | 49.41 | 38.018 | 0.018 | 0.51 | -642.994 |
|  | 5 | -820.821 | 138.515 | 62.39 | 48 | 0 | 0.471 | -642.994 |
| 1 year | 1 | -686.046 | 69.676 | 56.993 | 55.282 | 47.282 | 1 | -538.183 |
|  | 2 | -664.098 | 53.772 | 25.236 | 21.386 | 3.386 | 0.709 | -523.057 |
|  | 3 | -663.138 | 79.844 | 35.454 | 29.466 | 1.466 | 0.583 | -522.591 |
|  | 4 | -662.414 | 106.388 | 46.145 | 38.018 | 0.018 | 0.758 | -522.591 |
|  | 5 | -662.405 | 134.362 | 58.266 | 48 | 0 | 0.453 | -522.591 |
| 2 year | 1 | -633.616 | 76.07 | 63.39 | 61.953 | 53.953 | 1 | -497.67 |
|  | 2 | -608.294 | 53.073 | 24.542 | 21.308 | 3.308 | 0.529 | -476.703 |
|  | 3 | -606.676 | 77.485 | 33.103 | 28.073 | 0.073 | 0.761 | -476.703 |
|  | 4 | -606.64 | 105.059 | 44.826 | 38 | 0 | 0.505 | -476.703 |
|  | 5 | -606.64 | 132.706 | 56.623 | 48 | 0 | 0.428 | -476.703 |
| 3 year | 1 | -569.126 | 101.068 | 88.391 | 87.368 | 79.368 | 1 | -448.19 |
|  | 2 | -535.684 | 61.309 | 32.786 | 30.485 | 12.485 | 0.566 | -422.82 |
|  | 3 | -531.127 | 79.321 | 34.951 | 31.371 | 3.371 | 0.753 | -422.785 |
|  | 4 | -530.355 | 104.902 | 44.685 | 39.827 | 1.827 | 0.83 | -422.785 |
|  | 5 | -529.441 | 130.199 | 54.136 | 48 | 0 | 0.574 | -422.785 |
| 4 year | 1 | -467.838 | 80.417 | 67.748 | 67.493 | 59.493 | 1 | -379.174 |
|  | 2 | -440.695 | 52.286 | 23.78 | 23.206 | 5.206 | 0.65 | -359.356 |
|  | 3 | -439.098 | 75.248 | 30.904 | 30.013 | 2.013 | 0.844 | -358.839 |
|  | 4 | -438.091 | 99.391 | 39.21 | 38 | 0 | 0.604 | -358.839 |
|  | 5 | -438.091 | 125.547 | 49.529 | 48 | 0 | 0.521 | -358.839 |
| 5 year | 1 | -270.689 | 72.552 | 59.912 | 61.75 | 53.75 | 1 | -211.734 |
|  | 2 | -245.738 | 46.151 | 17.711 | 21.847 | 3.847 | 0.711 | -195.255 |
|  | 3 | -244.139 | 66.457 | 22.216 | 28.65 | 0.65 | 0.787 | -195.255 |
|  | 4 | -243.814 | 89.309 | 29.268 | 38 | 0 | 0.644 | -195.255 |
|  | 5 | -243.814 | 112.812 | 36.971 | 48 | 0 | 0.485 | -195.255 |

BIC: Bayesian Information Criterion, aBIC: adjusted BIC, AIC: Akaike’s Information Criterion

**Supplementary table 3 Fit statistics for latent transition analysis**

| Latent class | Measurement invariance across time | Loglikelihood | BIC | AIC | G-SQUARED |
| --- | --- | --- | --- | --- | --- |
| 2 | variance | -3164.87 | 3508.33 | 3285.81 | 3167.81 |
|  | invariance | -3186.79 | 3321.3 | 3249.64 | 3211.64 |
| 3 | variance | -3078.59 | 3595.48 | 3203.25 | 2995.25 |
|  | invariance | -3118.21 | 3328.43 | 3162.48 | 3074.48 |
| 4 | variance | -3014.16 | 3784.05 | 3184.39 | 2866.39 |
|  | invariance | -3066.11 | 3426.24 | 3128.29 | 2970.29 |
| 5 | variance | -2963.58 | 4058.04 | 3213.23 | 2765.23 |
|  | invariance | -3027.69 | 3609.1 | 3141.44 | 2893.44 |

BIC: Bayesian Information Criterion, AIC: Akaike’s Information Criterion

**Supplementary table 4 Variables for latent class analysis performed annually from baseline**

|  | Baseline | 1 year | 2 year | 3 year | 4 year | 5 year |
| --- | --- | --- | --- | --- | --- | --- |
| Pain |  |  |  |  |  |  |
| n | 321 | 270 | 252 | 227 | 187 | 110 |
| mean±sd | 3.3±2.8 | 2.8±2.8 | 2.8±2.7 | 2.7±2.8 | 2.9±2.8 | 2.5±2.8 |
| median(q1,q3) | 3(0,5) | 2(0,5) | 2(0,5) | 2(0,5) | 3(0,5) | 1(0,5) |
| Fatigue |  |  |  |  |  |  |
| n | 321 | 270 | 252 | 227 | 187 | 110 |
| mean±sd | 5.5±2.4 | 5.0±2.4 | 4.8±2.3 | 5.0±2.2 | 4.9±2.4 | 4.7±2.6 |
| median(q1,q3) | 5(5,7) | 5(4,7) | 5(3,7) | 5(3,7) | 5(3,7) | 5(3,7) |
| Dryness |  |  |  |  |  |  |
| n | 321 | 270 | 252 | 227 | 187 | 110 |
| mean±sd | 7.0±2.2 | 6.5±2.1 | 6.4±2.1 | 6.3±2.2 | 6.5±2.4 | 6.4±2.4 |
| median(q1,q3) | 7(5,8) | 7(5,8) | 7(5,8) | 7(5,8) | 7(5,8) | 7(5,8) |
| Anxiety/depression |  |  |  |  |  |  |
| n | 321 | 270 | 252 | 227 | 187 | 110 |
| mean±sd | 2.1±0.9 | 1.9±0.8 | 2.0±0.8 | 1.9±0.8 | 1.9±0.8 | 2.0±0.9 |
| median(q1,q3) | 2(1,3) | 2(1,2) | 2(1,2) | 2(1,2) | 2(1,2) | 2(1,2) |

Sd: standard deviation
